# Two Cases With an Early Presented Proopiomelanocortin Deficiency—A Long-Term Follow-Up and Systematic Literature Review

**DOI:** 10.3389/fendo.2021.689387

**Published:** 2021-06-09

**Authors:** Nadan Gregoric, Urh Groselj, Natasa Bratina, Marusa Debeljak, Mojca Zerjav Tansek, Jasna Suput Omladic, Jernej Kovac, Tadej Battelino, Primoz Kotnik, Magdalena Avbelj Stefanija

**Affiliations:** ^1^ Department for Endocrinology, Diabetes and Metabolic Diseases, Division of Internal Medicine, University Medical Centre Ljubljana, Ljubljana, Slovenia; ^2^ Faculty of Medicine, University of Ljubljana, Ljubljana, Slovenia; ^3^ Department of Endocrinology, Diabetes and Metabolic Diseases, University Children’s Hospital, University Medical Centre Ljubljana, Ljubljana, Slovenia; ^4^ Clinical Institute for Special Laboratory Diagnostics, University Children’s Hospital, University Medical Centre Ljubljana, Ljubljana, Slovenia

**Keywords:** proopiomelanocortin, POMC deficiency, obesity, adrenal insufficiency, type 1 diabetes, setmelanotide, systematic review

## Abstract

Proopiomelanocortin (POMC) deficiency is an extremely rare inherited autosomal recessive disorder characterized by severe obesity, adrenal insufficiency, skin hypopigmentation, and red hair. It is caused by pathogenic variants in the *POMC* gene that codes the proopiomelanocortin polypeptide which is cleaved to several peptides; the most notable ones are adrenocorticotropic hormone (ACTH), alpha- and beta-melanocyte-stimulating hormones (*α*-MSH and *β*-MSH); the latter two are crucial in melanogenesis and the energy balance by regulating feeding behavior and energy homeostasis through melanocortin receptor 4 (MC4R). The lack of its regulation leads to polyphagia and early onset severe obesity. A novel MC4R agonist, setmelanotide, has shown promising results regarding weight loss in patients with POMC deficiency. A systematic review on previously published clinical and genetic characteristics of patients with POMC deficiency and additional data obtained from two unrelated patients in our care was performed. A 25-year-old male patient, partly previously reported, was remarkable for childhood developed type 1 diabetes (T1D), transient growth hormone deficiency, and delayed puberty. The second case is a girl with an unusual presentation with central hypothyroidism and normal pigmentation of skin and hair. Of all evaluated cases, only 50% of patients had characteristic red hair, fair skin, and eye phenotype. Central hypothyroidism was reported in 36% of patients; furthermore, scarce adolescent data indicate possible growth axis dysbalance and central hypogonadism. T1D was unexpectedly prevalent in POMC deficiency, reported in 14% of patients, which could be an underestimation. POMC deficiency reveals to be a syndrome with several endocrinological abnormalities, some of which may become apparent with time. Apart from timely diagnosis, careful clinical follow-up of patients through childhood and adolescence for possible additional disease manifestations is warranted.

## Introduction

Proopiomelanocortin (POMC) is a precursor polypeptide hormone secreted primarily in the hypothalamus. The post-translational process and cleavage give rise to several polypeptides, the melanocortins. Among these are adrenocorticotropic hormone (ACTH), alpha-, beta- and gamma-melanocyte-stimulating hormones (*α*-MSH, *β*-MSH, and *γ*-MSH), *β*-lipotrophin, and endorphins (1). These polypeptides bind to melanocortin receptors of different subtypes. The melanocortin 1 receptor (MC1R) regulates skin pigmentation; stimulation of MC2R induces adrenal steroidogenesis; MC3R and MC4R regulate energy balance through appetite regulation, whereas MC5R is expressed in sebaceous glands and is involved in sebogenesis (1). To add to the complexity, the POMC neurons in the central nervous system are highly heterogeneous in their regulation and action; furthermore, the POMC-derived peptides can have opposing effects on appetite regulation, *α*-MSH suppressing and *β*-endorphin, on the other hand, promoting appetite (2).

Major insights into POMC function in humans are derived by studying patients with POMC deficiency (OMIM#609734). Biallelic loss-of-function variants in the *POMC* gene give rise to a phenotype with a triad of clinical features: ACTH deficiency that is usually the first to be recognized, hypopigmented skin with red hair, and early onset obesity due to uncontrolled polyphagia. The cornerstone of treatment is glucocorticoid replacement and weight management, the latter being very challenging. Novel treatment, such as MC4R agonist, setmelanotide, has proven to be effective in reducing and maintaining body weight (3).

The condition is extremely rare; only a handful of cases have been reported since the first two cases have been described in 1998 (4). We gathered and analyzed the current experience on POMC deficiency by performing a systematic review of the literature. To increase the amount of information, we also included detailed clinical data on two unrelated patients with POMC deficiency from our center, a 25-year-long follow-up of a male patient with type 1 diabetes and a 4-year follow-up of a female patient with an unusual phenotype. It appears that POMC deficiency could also have important clinical consequences outside the classical triad, some of which could become apparent with time.

## Methods

The clinical information of the two patients who have been followed regularly at the University Medical Centre Ljubljana, Slovenia was gathered from the medical documentation. Written informed consent using local consent forms were obtained from patient 1 and parents of patient 2 for the publication of any potentially identifiable images or data included in this article. The pubertal stage was evaluated by a trained pediatric endocrinologist; testicular volume was estimated using a prader orchidometer. Anthropometric measurements were performed by trained nurses using professional certified digital devices. Gonadotropin-releasing hormone (GnRH) stimulation test was performed using gonadorelin (Relefact LH–RH, Sanofi-Aventis, Germany) 100 µg/m^2^ body surface intravenously; blood samples were taken at 0, 20, 30, and 60 min, and luteinizing hormone (LH) and follicle-stimulating hormone (FSH) were measured by immunoassay using Immulite 2000 (Siemens, Germany). Other dynamic tests, including arginine, levodopa (L-DOPA) growth hormone (GH) stimulation tests, oral glucose tolerance test, and standard and low-dose Synachten tests, were performed according to previously published test procedures (5). The Whole-Body Insulin Sensitivity Index (WBISI) was calculated as previously described (6). Genetic analysis of Case 1 was reported previously (7). In Case 2, genetic analysis was performed after obtaining informed consent approved by the Republic of Slovenia National Medical Ethics Committee (#132/03/15). Next-generation sequencing (NGS) was performed. The regions of interest were enriched using TruSight One library enrichment kit (Illumina, San Diego, CA, United States) according to the manufacturer’s instructions and sequenced on the MiSeq desktop sequencer together with MiSeq Reagent kit v3 (Illumina, San Diego, CA, United States). A panel of 59 genes associated with obesity, including the *POMC* gene, was used for filtering of variants. The variant identified was subsequently confirmed by Sanger sequencing.

**Figure 1:**
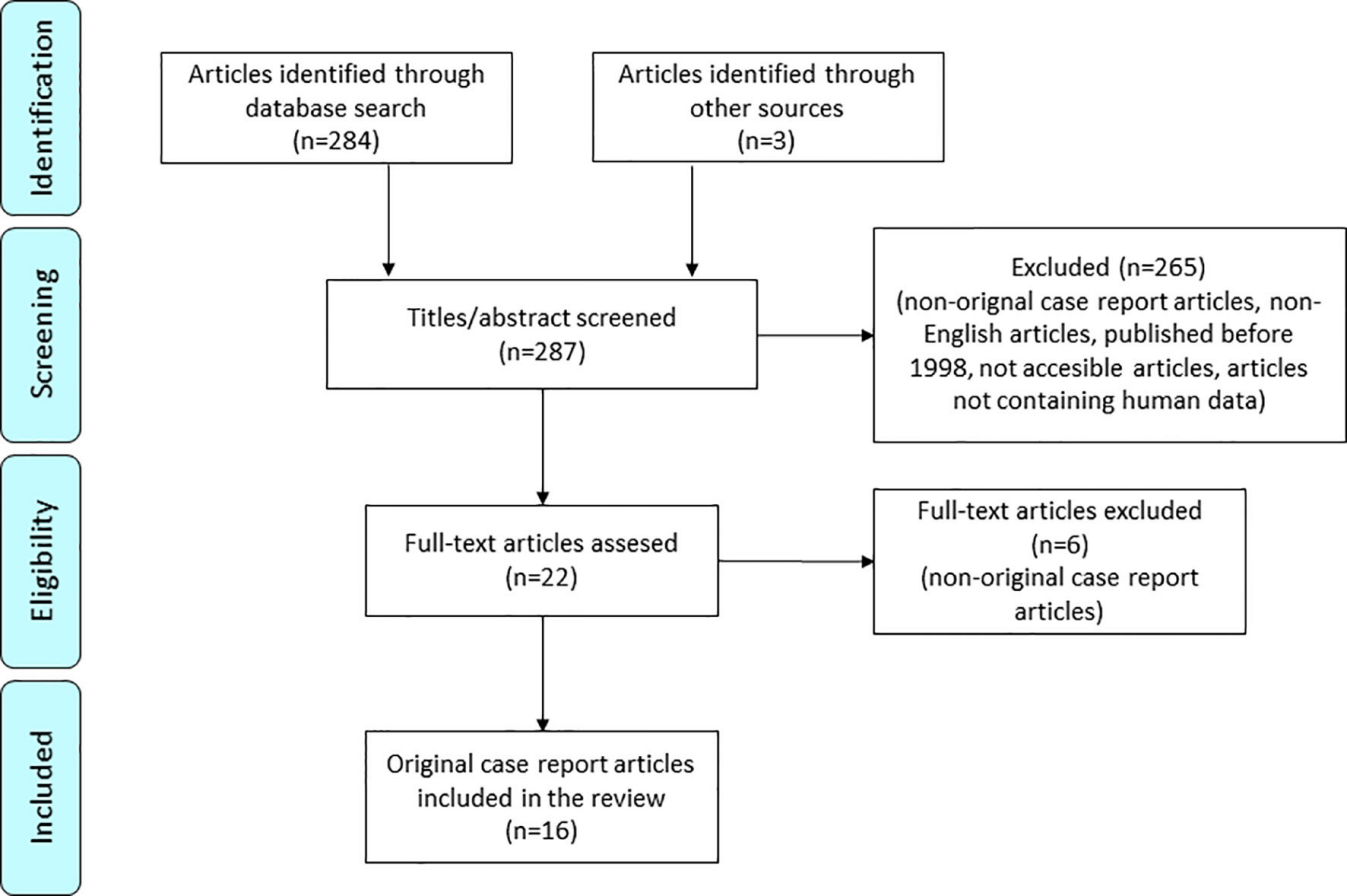
PRISMA flow diagram for systematic case review of POMC deficiency (8).

For the systematic review, we collected all the available scientific case report articles on POMC deficiency (**Figure 1**). The following search terms were used: “POMC” (AND) “deficiency”. We found 287 research articles. By reading all the abstracts and titles, we excluded articles that did not meet the following conditions (i): articles in English published after 1998, when POMC deficiency was described for the first time; (ii) articles that were fully accessible; (iii) articles containing human data; (iv) original case report articles. Additional case reports were found through the articles’ reference list. In the end, 16 articles and newly reported clinical data of our two patients were included.

## Results

### Case Description Number 1

**Figure 2:**
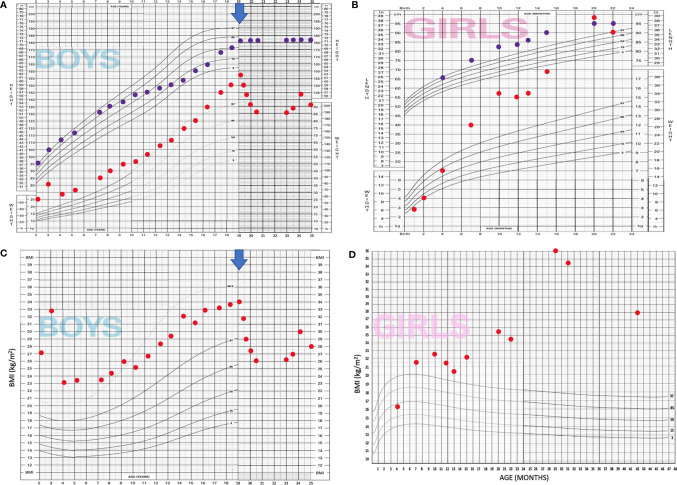
Modified WHO growth charts* display both case patients. **(A)** Case patient 1—length for age and weight for age; **(B)**: Case patient 1—BMI for age, **(C)**: Case patient 2—length for age and weight for age, **(D)**: Case patient 2—BMI for age; BLUE ARROW marks the onset of setmelanotide treatment of case patient 1. *Original WHO growth charts were modified due to restrictions of scale and age.

**Figure 3:**
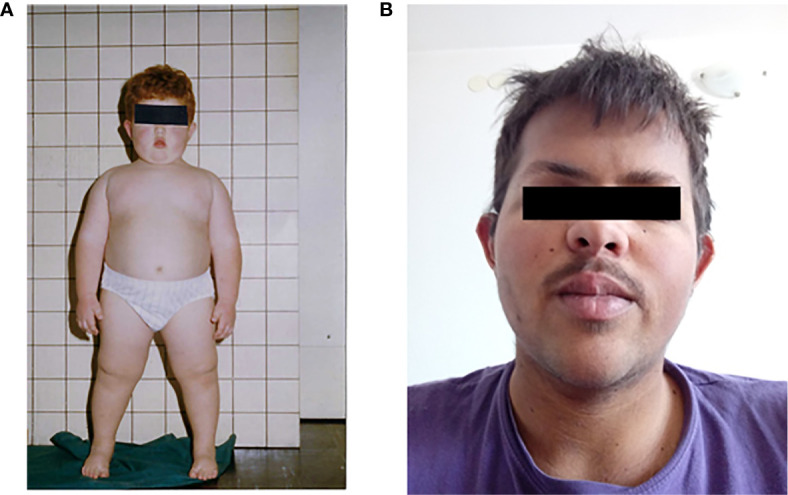
Case patient 1 in childhood, before setmelanotide treatment **(A)** and after **(B)**.

A male patient (#5 in **Table 1**), partially described in previous publications (3, 7), was diagnosed with POMC deficiency caused by compound heterozygous variants c.151A>T and c.296del. The selected laboratory data is demonstrated in **Table 2**. He was born at term after an uneventful pregnancy to non-consanguineous parents, heterozygous carriers. His birth weight was 3,300 g. Neonatally, he developed hypoglycemia with convulsions and was treated in an intensive care unit where he required mechanical ventilation and parenteral feeding. Due to extremely low cortisol concentrations and ACTH levels, he was diagnosed with central adrenal insufficiency and put on substitutional treatment with hydrocortisone. No thyroid axis dysfunction was identified. The ultrasound and MRI later revealed atrophy of adrenal glands. Despite being treated with a low dose of hydrocortisone, his body weight rapidly increased due to uncontrollable polyphagia. At 24 months, he weighed 25 kg. Besides adrenal insufficiency and early onset obesity, another prominent physical feature was extremely pale skin and red hair that was in contrast to his immediate family members. His father was overweight; mother had normal weight. The patient followed a restriction diet program which success was only short-lived; the patient continued to gain weight (**Figure 2A**) until 7 years of age when he lost 4 kg due to symptomatic hyperglycemia and was hospitalized due to diabetic ketoacidosis. His C-peptide and insulin levels were low, and he was diagnosed with type 1 diabetes. The autoantibodies were not determined. In the following years, he was repeatedly hospitalized due to diabetes-related complications (severe hypoglycemia and diabetic ketoacidosis). Despite extensive efforts, the glycemia remained challenging due to poor diabetes management driven by behavioral and learning difficulties and uncontrolled hyperphagia. The patient often intentionally induced hypoglycemia in order to get an extra meal. Due to extreme obesity and high insulin requirement, indicating insulin resistance, he was treated with metformin 850 mg BID for a decade until 18 years of age, but the treatment failed to provide any significant effect. For a brief period, he was treated with appetite suppressant sibutramine. He lost 2 kg of body weight in 2 months but the success was short-lived as the drug was soon removed from the market. After the age of 20, he was receiving glucagon-like peptide-1 receptor agonist, liraglutide, titrated to 1.8 mg QD, again failing to produce any significant weight reduction or appetite suppression. At the age of 22 years, he was enrolled in the phase 3 trial with a novel MC4R agonist, setmelanotide (3). The trial lasted 12 months. During the trial, the polyphagia was well controlled, and the patient was able to lose approximately 27 kg of body weight from 113 kg (BMI 34 kg/m^2^) to 86 kg (BMI 26 kg/m^2^), demonstrated in **Figures 2A, B**. As the treatment continued, the patient was mostly able to maintain the body weight and curb the appetite. It is noteworthy that during the setmelanotide treatment, his once pale skin and red hair gradually became hyperpigmented. At the time of writing this paper, he had dark brown hair and brown skin color as demonstrated in **Figure 3**. His blue eyes became brown.

**Table 1:** A list of reported cases of POMC deficiency so far.

Patient	Nucleotide change	AA change	Ancestry	Gender	Age of first symptoms	First presenting symptoms/signs	Hair color	Other endocrine comorbidities	Reference
1	c.313G>T c.433delC	p.Glu105* p.Arg145fs	German	male	neonatal	hyperbilirubinemia	red	subclinical central hypothyroidism	(4)
2	c.-11C>A		German	female	12 months	hypoglycemia, hyponatremia	red	subclinical central hypothyroidism, GH deficiency, hypogonadism	(4, 7, 9)
3	c.-11C>A		Dutch	male	neonatal	hypoglycemia, convulsions, hyperbilirubinemia	red, but changed to brown at 2–3 years		(7)
4	c.-11C>A/c.403_404dupGG	p.Glu134fs	Swiss	female	6 months	hypoglycemia, convulsions	red		(7)
5	c.151A>T c.296delG	p.Lys51* p.Gly99fs	Slovenian	male	neonatal	hypoglycemia, convulsions	red	type 1 diabetes, GH deficiency, hypogonadism	(3, 7), this report
6	c.206delC	p.Pro69fs	Turkish	male	not specified	not specified	brown, but dark red roots		(10)
7	c.223dupC	p.Arg75fs	North African	female	4 weeks	hypoglycemia	brown	GH deficiency, hypogonadism, central hypothyroidism	(9, 11)
8	c.296delG	p.Gly99fs	Turkish	male	neonatal	hypoglycemia, apnea attacks	red	mineralocorticoid deficiency	(12)
9	c.231C>A	p.Tyr77*	Hispanic	female	9 months	hypoglycemia, hyponatremia	dark brown to black		(13)
10	c.256C>T	p.Arg86*	Indian	male	neonatal	respiratory distress, convulsions, hypoglycemia, hyponatremia	skin and hair lighter than expected	central hypothyroidism	(14)
11	c.202C>T	p.Gln68*	Egyptian	male	neonatal	hypoglycemia	dark brown to black		(15)
12	c.206delC	p.Pro69fs	Turkish	male	neonatal	convulsions, apnea	red, but brown later	central hypothyroidism	(16)
13	c.-11C>A c.433C>T	p.Arg145Cys	French-Canadian	female	4.3 years	hypoglycemia, hyponatremia	red	elevated bioinactive ACTH	(17)
14	c.433C>T	p.Arg145Cys	French-Canadian	male	4 months	hypoglycemia, convulsions, hyponatremia	red	elevated bioinactive ACTH	(17)
15	c.-11C>A		Scottish/German	male	neonatal	hypoglycemia, convulsions	red	type 1 diabetes	(18)
16	c.64delA	p.Met22fs	Turkish	female	neonatal	hyperbilirubinemia, hypoglycemia, convulsions	red		(19)
17	c.-11C>A c.251G>A	p.Trp84*	Russian	male	neonatal	hyperbilirubinemia, hypoglycemia	red	subclinical hypothyroidism	(20)
18	c.133-2A>C		Iraqi	female	neonatal	hyperbilirubinemia, frequent falls	light brown with a reddish hue	type 1 diabetes	(21)
19	c.133-2A>C		Iraqi	female	neonatal	hyperbilirubinemia	light brown with reddish hue		(21)
20	c.20_21ins25	p.Ser7fs	Hispanic	male	neonatal	hypoglycemia, hyperbilirubinemia, poor feeding	dark with a reddish tinge	central hypothyroidism	(22)
21	c.206delC	p.Pro69fs	Turkish	female	2.5 months	spasms, cyanosis, hypoglycemia, hyponatremia, elevated aspartate transaminase	red	no mini-puberty	(23)
22	c.296delG	p.Gly99fs	Slovenian	female	7 months	obesity, central hypothyroidism	brown	central hypothyroidism	this report

The reference numbers of POMC gene and POMC protein are NM_001035256.3 and NP_001030333.1, respectively. AA, amino acid; GH, growth hormone; ACTH, corticotropin.

**Table 2:** Laboratory values of case patient 1.

Biochemistry/Age	3 months	2.0 years	3.0 years	5.5 years	7 years	13.5 years	14.5 years	15 years	15.5 years	16.3 years	25 years
**TSH [mU/L]** **(0.59–4.23)**	3.83			NA	3.912	2.281	1.584			2.54	4.5
**fT4 [pmol/L]** **(11.7–22.5)**	14.9			16.5	13.2					13.57	16.0
**fT3 [pmol/L]** **(3.79–6.05)**	4.0			7	5.3					5.23	7.1
**IGF-1 [μg/L]**				312 (high)		141	103	89.4			128
**IGFBP3 [mg/L]**						4.6	5.02	5.89			2.67
**GH (basal/peak) [μg/L]**				2.9			0.199/2.76 (arginine)	0.515/1.76 (L-DOPA)			
**LH [U/L]**						<0.1	0.665	0.566		1.32	4.6
**FSH [U/L]**						0.919	2.2	2.27		1.64	2.2
Cortisol (basal/peak)* [nmol/L]	40.7/54	12/21	171^†^								
**ACTH [pmol/L]** **(<10.20)**			<2.2								
**PRA [μg/L/h]**				0.24 (low)	0.54						
**Insulin (basal/2 h) [mU/L]**		4.3		2.5/37.4							
**Glucose (basal/2 h) [mmol/L]**		3.7	3.4	3.1/7.0	22.7						
**Androstendion [nmol/L]** **(0.7–10.8)**											1.47
**DHEA-S [μmol/L]** **(3.6–12.9)**							0.0				1.6
**SHBG [nmol/L]** **(18–114)**											67.0
**Testosterone, total [nmol/L]**						0.0	0.0	0.05		2.5	33.7
**Testosterone, free [pmol/L]**											64.4
**Total cholesterol** **[mmol/L] (<5.2)**		4.1	4.5	4.2	3.2	5.6	4.1			6.1	4.5
**LDL [mmol/L] (<3.4)**		2.5	2.7	2.6	1.9	3.0	2.7			3.1	2.9
**HDL [mmol/L] (>1.3)**		0.5	0.9	1.0	0.7	0.7	0.7			0.8	1.0
**Triglycerides [mmol/L] (<1.7)**		2.1	2.0	1.2	1.4	4.0	2.8			10.7	1.4
**Pubertal status**				A1, P1		A1, P1	A1, P1	A1, P1	P2	A1, P2	
**Testicular volume [mL]**				1-2	2	2	3	1-2	8-10	15	

Reference values are stated in brackets. *Peak values were obtained 60 minutes after stimulation with synthetic ACTH 125 µg and 250 µg intramuscularly at 3 months and 2 years, respectively, ^†^value obtained while on substitution with hydrocortisone.

During the first year of life, he suffered a couple of complex febrile convulsions. His psychomotor development was delayed; he began walking at 22 months, but gradually caught up. Later, as he acquired type 1 diabetes, he experienced very frequent hypoglycemic seizures, occasionally without noting symptoms of preceding hypoglycemia. The electroencephalography did not show any epileptiform activity.

In his teens, there was a significant growth delay, a drop from 95 percentile to 10th percentile in 5 years (**Figures 2A, B**). Further evaluation revealed GH deficiency, which was confirmed by arginine and L-DOPA GH stimulation tests and a decrease in insulin-like growth factor-1 (IGF-1) levels, which were, in childhood, above the normal range. The bone age was significantly delayed, 9 years, 4 months (−5.3 *σ*) at 14 years of age. Testosterone levels were low, and testicular volume started to develop spontaneously at 14–15 years up to 15 ml. He developed very poor body, pubic, and axillary hair, and adrenal androgens remained low into adulthood. A GH substitutional therapy was considered but never commenced as growth spurt occurred along with the slightly delayed puberty. His final height was 183 cm. It was only around 22 years of age, on setmelanotide for a year, that the patient developed notable body hair (pubic and chest hair) and began shaving his beard. Recently, at the age of 25, we evaluated his hypothalamic–pituitary–gonadal (HPG) axis and demonstrated normal function with high-normal testosterone levels. IGF-1 was within normal limits.

At the age of 15, he was evaluated for respiratory acidosis and narcolepsy and was diagnosed with obstructive sleep apnea.

In early childhood, he was also diagnosed with a small restrictive muscular ventricular septal defect that was persistent but did not require treatment.

### Case Description Number 2

A 4-year-old girl (#22 in **Table 1**) who was born as a third child to distantly related parents, members of the Romani ethnic minority, was diagnosed with POMC deficiency caused by a homozygous frameshift variant in POMC (NM_000939.2:c.296delG). She was born at 35 weeks of gestation, with birth weight and length between the 10th and 50th percentiles. She had a complicated neonatal period. Paroxysmal tachycardia was noted already during pregnancy. After birth, paroxysmal intra-atrial tachycardia was defined, atrial undulation, and consequently decreased cardiac systolic function. She started therapy with amiodarone and metoprolol and needed cardioversion. Arrhythmia resolved completely in the first few months of life. Transiently, a small atrial septal defect type secundum and a small perimembranous ventricular septal defect were noted.

She had signs of necrotizing enterocolitis, which was treated conservatively. During the first month, she had problems with obstipation; the feeding was difficult due to drowsiness. Hirschsprung disease was excluded by biopsy. The symptoms disappeared in the next months.

Neonatally, she had a prolonged requirement of phototherapy for indirect hyperbilirubinemia that started on the second day of life; slightly elevated was also conjugated bilirubin, which was attributed to ABO alloimmunization. No hypoglycemia was noted. Adrenal glands had a normal ultrasound appearance, and adrenal function at that time was not evaluated.

Since birth, she had suboptimal neurological development, delayed motor milestones, and muscular hypotonia. Brain imaging neonatally showed increased periventricular echogenicity, mild corpus callosum hypoplasia, mildly wider lateral ventricles, and signs of intracerebral micro bleedings, which were attributed to perinatal complications. By brain magnetic resonance imaging (age 1.5 years), slightly smaller pituitary and lower part of infundibulum were noted.

For the follow-up of thyroid function on amiodarone treatment, she came to the attention of an endocrinologist. The selected biochemical results are summarized in **Table 3**.

**Table 3:** Laboratory values of case patient 2.

Biochemistry/Age	9 days	3 weeks	7 months	11 months	20 months	2.5 years	3.5 years
**TSH [mU/L]** **(0.59–4.23)**	3.83	7.84	5.34	0.92*		3.24*	3.90*
**fT4 [pmol/L]** **(11.7–22.5)**	14.9	15.0	8.2	14.8*		11.3*	14.9*
**fT3 [pmol/L]** **(3.79–6.05)**	4.0	4.8	5.9	6.7*		5.74*	4.86*
**IGF-1 [μg/L]**				100 (20–159)	213 (19.5–132.3)	174 (22.2–145.4)	186 (25.9–164.2)
**IGFBP3 [mg/L]**				4.48 (1.37–4.30)	4.49 (1.22–3.72)	6.10 (1.39–4.15)	4.40 (1.55–4.56)
**GH (basal/peak)† [μg/L]**				0.73/3.49		0.34	
**LH (basal/peak) [U/L]**				0.2/8.5			
**FSH (basal/peak) [U/L]**				5.2/26.3			
**Cortisol (basal/peak)‡ [nmol/L]**				<27.5/<27.5			
**ACTH [pmol/L]** **(<10.20)**				<1.11			
**PRA [μg/L/h]**				8.72			
**Insulin (basal/2 h)§ [mU/L]**				9.4			3.5/238.0
**Glucose (basal/2 h)§ [mmol/L]**				4.2			4.0/6.7
**AST [μkat/L]** **(<0.52)**						1.25	0.55
**ALT [μkat/L]** **(<0.57)**						1.09	0.78
**ϒGT [μkat/L]** **(<0.63)**						1.40	0.21
**Androstendion [nmol/L]** **(0.7–10.8)¶**				<0.1		<0.1	
**DHEA-S [μmol/L]** **(3.6–12.9)¶**				0.09		<0.08	
**SHBG [nmol/L]** **(18–114)¶**						29	
**17-OHP [nmol/L]**						0.17	
**Total cholesterol** **[mmol/L] (<5.2)**							2.8
**LDL [mmol/L] (<3.4)**							1.7
**HDL [mmol/L] (>1.3)**							0.8
**Triglycerides [mmol/L] (<1.7)**							0.7

*thyroid function with L-thyroxin supplementation; † Peak value was obtained after stimulation with arginine; ‡ Peak value was obtained 30 min after intravenous stimulation with synthetic ACTH 1 µg; § values were obtained with oral glucose tolerance test; ¶ normal range in adults. The reference values are stated in brackets.

She had a normal thyroid function at age 9 days, mild latent primary hypothyroidism on amiodarone therapy at 3 weeks, while central hypothyroidism was identified at the age of 7 months after cessation of amiodarone and levothyroxine substitution. Subsequently, central hypocorticism was diagnosed in an asymptomatic state. She had elevated growth factors but peak stimulated GH at single testing was subnormal. She was, however, tall for her age. At 11 months, gonadotropins were appropriate for age.

She had normal weight at the age of 4 months (**Figures 2C, D**); at the age of 2.7 years she had an exponential weight gain up to 43 kg (height 111 cm, BMI 34.5 kg/m^2^, +5.24 SDS) despite multiple dietary counseling. At 3.4 years, with improved parental control, she lost some weight (BMI 27.76 kg/m^2^, +3.84 SDS). She had ultrasound signs of hepatic steatosis at the age of 2.7 years, elevated liver transaminases, and decreased HDL cholesterol. By the age of 3.5 years, she had normal glucose tolerance and HOMA index 0.6 but markedly elevated stimulated insulin, decreased WBISI 0.44, and acanthosis nigricans, indicative of insulin resistance. Simultaneously she had early signs of alveolar hypoventilation at nocturnal polysomnography. Interestingly, she had dark brown hair and eyes.

At the time of publication, she was receiving levothyroxine and hydrocortisone.

### Review of Previously and Currently Reported Cases

All 22 reported cases, including our cases 1 and 2, are summarized in **Table 1**. All POMC deficient patients had early onset obesity and adrenal insufficiency, the latter being the first diagnosis to be established. The most common presenting sign, reported in 16 of 22 cases, was neonatal hypoglycemia with or without convulsions that usually prompted further workup that led to the diagnosis of adrenal insufficiency. It is worth noticing that in eight of 22 cases there was a preceding hyperbilirubinemia which may signify impending liver failure due to adrenal insufficiency. In five cases there were reports of siblings who had died from liver failure or sepsis within a few months of life (4, 10, 11, 21, 22). A *postmortem* analysis confirmed POMC deficiency in one of these cases (4), which suggests that some patients may succumb to complications of untreated adrenal insufficiency before the diagnosis of POMC deficiency is even made.

Due to lack of uniformity in case presentations, there is a considerable discrepancy in reported clinical features with a possibility of under-reporting, which is one of the limitations of this review article. The most notable discrepancy is the presenting age of signs or symptoms, ranging from the neonatal period to 4.3 years. The majority of the cases report only on childhood; three cases also include adolescence, and of these only one, our case, extends to adulthood.

**Figure 4:**
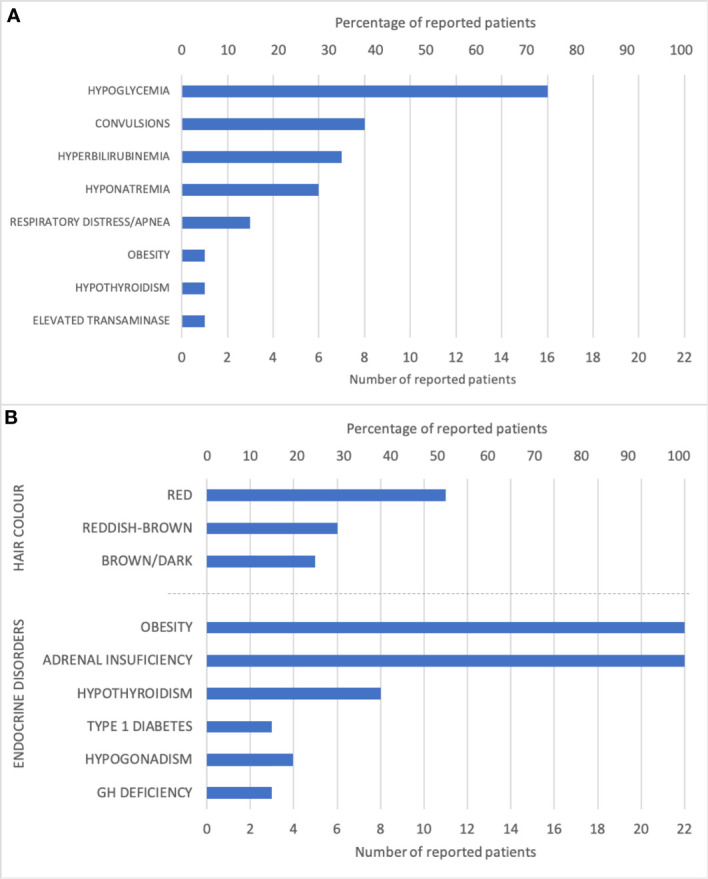
Graphic representation of **(A)** the frequency of first presenting signs or symptoms and **(B)** the frequency of clinical features.

Since present in all the cases early onset obesity and adrenal insufficiency were not included in the table. The summary of presenting symptoms and clinical characteristics is presented in **Figure 4**; however, the prevalence of characteristics that may appear with time could be underestimated due to the young age of patients having been reported.

**Figure 5:**
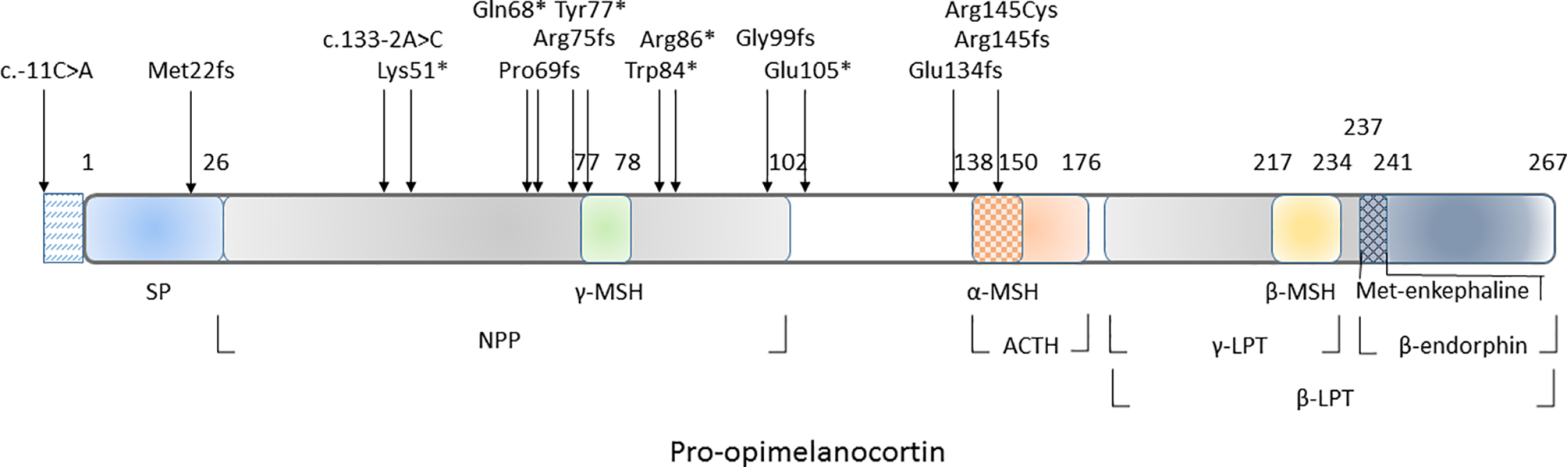
Schematic representation of POMC-derived peptides and genetic variants associated with autosomal recessive POMC deficiency. ACTH, corticotropin; *β*-LPT, lipotropin *β*; *γ*-LPT, lipotropin *γ*; *α*-MSH, melanotropin *α*; *β*-MSH, melanotropin *β*; *γ*, MSH, melanotropin *γ*; NPP, N-terminal peptide of proopiomelanocortin; SP, signal peptide.

All genetic variants associated with autosomal recessive POMC deficiency are listed in **Table 1** and marked in the *POMC* gene schematic in **Figure 5**.

## Discussion

POMC deficiency is an ultra-rare syndrome. At the time of writing this paper, there have been only 22 cases reported in the literature, including our two cases. Despite its extreme rarity, it should not be considered a mere exotic oddity. It is a treatable condition with three distinct features that enable early diagnosis. Adrenal insufficiency, early onset obesity, and red hair/fair skin are the most common and recognizable features, but as demonstrated in this review, there is a high variability of the phenotype that is often supplemented by additional endocrine disorders such as central hypothyroidism, hypogonadotropic hypogonadism, and type 1 diabetes that shed a new perspective on the pathophysiology of POMC deficiency. Furthermore, our long-term follow-up of a patient with POMC deficiency yielded some interesting insights into the natural course of the disease through the period of adolescence and early adulthood.

Present in all the cases, adrenal insufficiency is usually the first clinical feature and often occurs neonatally with hypoglycemia, hyperbilirubinemia, or signs of liver failure (**Table 1** and **Figure 4A**). The diagnosis of adrenal insufficiency, in most cases, is established within the first year, although in some cases, the clinical features are not evident, and the diagnosis can be delayed, even up to 9 years (21). The weight gain is independent of hydrocortisone replacement treatment. Newborns are usually of normal weight but gain weight at an accelerated pace and develop extreme obesity within a year. The infants are hyperphagic with uncontrollable hunger. At this point, POMC deficiency is very likely, and diagnosis should be considered.

The presence of red hair and fair skin makes the probability of diagnosis even more likely. The characteristic pigment changes in POMC deficiency are caused by the absence of MC1R stimulation by *α*-MSH (24, 25). When stimulated, MC1R shifts the expression of pheomelanin (red pigment) towards eumelanin (dark pigment). While *α*-MSH acts as the main MC1R agonist, Agouti-related protein (AgRP) acts as an MC1R antagonist (26). In reported POMC deficient patients (**Table 1** and **Figure 4B**), the whole spectrum of pigmentation phenotype can be observed. It ranges from characteristic red hair, fair skin, and eye phenotype described only in 11 out of 22 patients (50%), lighter than expected hair or reddish-brown hair (27%), up to dark brown hair and eyes described in five patients including our female patient (23%) (10, 11, 13, 15). Previously reported hair pigment analyses in the dark-haired patients demonstrated a markedly increased amount of pheomelanin and pheomelanin/eumelanin ratio (11, 15), signifying unopposed AgRP action on MC1R. Interestingly, eumelanin was either close to normal (15) or even elevated (11), likely a result of a constitutive, *α*-MSH independent MC1R signalization that was previously demonstrated in mice and humans (27, 28). While the complete deficiency of POMC results in the absence of all melanocortins and should provide a phenotype with red hair and fair skin, other gene variants that still permit the synthesis of *γ*-MSH might provide enough MC1R stimulation to allow some pigmentation. Despite this residual MC1R stimulation, these patients still have adrenal insufficiency since they lack ACTH which is the only ligand for MC2R that is present only in the adrenal cortex. In our dark-haired girl, the genetic variant was located downstream of *γ*-MSH (**Figure 5**). Yet, this mutation was reported in another patient, which presented with red hair (12). Actually, in most dark-haired patients, even *γ*-MSH synthesis is abolished (9, 10, 13, 15, 22); dark hair color phenotype in these patients, therefore, seems not to result from any residual POMC-derived melanocortin. Furthermore, there are few other reports of patients displaying different phenotypes with either red or brown hair while having the same genotype (7, 10, 16, 23). The common denominator of patients with darker pigmentations seems to be a non-Caucasian ethnic origin, including our female patient that is of Romani descent. In clinical practice, therefore, one should not rely on pigmentation phenotype when confronted with a patient having other clinical signs of POMC deficiency. The skin phenotypes seem to depend on other inherited factors influencing skin pigmentation such as those characteristic of certain ethnic origins and probably associated with augmented constitutive MC1R signalization. Interestingly, setmelanotide, which is an MC4R agonist, increases pigmentation in all POMC deficient patients, including our male patient, but also in some leptin receptor (LEPR) and MC4R deficient patients and even in obese control subjects (4, 29). This observation likely at least partly results from a previously demonstrated ability of setmelanotide to also stimulate MC1R (29). However, human melanocytes express MC4R as well, whose stimulation increases melanogenesis (30).

Outside of the classical triad, other comorbidities are often found along with POMC deficiency. Most frequently occurring is subclinical central hypothyroidism, reported in eight out of 22 patients (36%) (7, 14, 16, 22). Whether there is a direct correlation between the absence of melanocortins and what predisposes a subset of patients to this phenotype remains to be determined. In our girl patient, hypothyroidism was not congenital, since she had confirmed adequate TSH secretion in the first months of life, while central hypothyroidism was evident by 7 months of age. A similar course of thyroid axis function was observed in the patient described by Hung et al. (14). The interrelationship between the hypothalamic–pituitary–thyroid (HPT) axis, nutrition, and energy balance has been well established. The thyrotropin-releasing hormone (TRH) secretion is normally reduced during starvation to reduce the basal metabolic rate to conserve fat and energy stores. The effect is then reversed by ingestion of food and surge of leptin (31), which stimulates the HPT axis and increases the energy consumption in an abundance of nutrients. It has been demonstrated that TRH secretion is stimulated by leptin directly and indirectly through melanocortin signaling pathways with binding the *α*-MSH to MC4R (32). Therefore, it would be expected to observe a well-stimulated HPT axis in obese hyperphagic individuals. Moderately elevated TSH values have indeed been consistently observed in obese populations where subclinical hypothyroidism has been excluded. It has been postulated that elevated thyroid hormone levels were an adaptive mechanism to increase the resting energy expenditure and reduce the conversion of energy into fat (33). Instead, in POMC deficiency, central hypothyroidism seems to be a common occurrence, and the absence of MC4R stimulation could very well be the culprit here. The diminished leptin effect on TRH stimulation is also evident in LEPR deficiency where central hypothyroidism is observed in 13% of cases (34). It would be interesting to observe whether the stimulation of MC4R with an agonist such as setmelanotide would improve central hypothyroidism to any extent, but unfortunately, there were no reports of such results in the literature to date.

The interrelation between melanotropins and somatotrophs is, to the best of our knowledge, unknown. Yet, in humans, MC4R deficiency is associated with increased height gain as compared to similarly obese control population, comparable and normal IGF-1 and IGF-2 values, and increased GH secretion (35). In MC4R knock-out mice, to the contrary, GH and IGF-1 suppression was observed recovered when hyperphagia was prevented and hyperinsulinemia reversed (36). Early growth acceleration was observed in both Slovenian patients (**Figure 2**) and is reported in all other case presentations. Nevertheless, GH deficiency (11) and decreased IGF-1 (9) were documented in three adolescents, including our male patient. While having increased IGF-1 in childhood, our patient had markedly decreased growth velocity at the time of anticipated puberty and confirmed GH deficiency. However, as the delayed puberty finally commenced, so did spontaneous growth acceleration. In childhood, the reported IGF-1 levels were within the normal range (13, 21, 22). As most patients were prepubertal when reported, the prevalence of growth hormone deficiency in adolescence in our review could be underestimated. Of note, setmelanotide therapy did not significantly affect IGF-1 levels in the two reported patients with GH deficiency, nor did a significant weight loss (9). On the contrary, after 3 years of setmelanotide treatment and significant weight loss, our male patient had a normal IGF-1.

Energy metabolism and reproduction are tightly linked, as the process of fertility requires proper energy reserves. Obesity, particularly when combined with insulin resistance and/or type 2 diabetes, is closely related to hypogonadotropic hypogonadism. There is an inverse correlation between free testosterone concentration and BMI as well as insulin resistance (37), and it is not restricted only to adult men. Even obese adolescent boys have 40% lower free testosterone concentrations compared to lean counterparts, and 40% of these obese individuals have subnormal testosterone concentrations (38). The circulating insulin and leptin levels, both deranged in POMC deficient patients (9), seemingly play a pivotal role in affecting the HPG axis (39). Currently, the most widely accepted mechanism of diabesity-related hypogonadism is that insulin and leptin resistance diminishes the stimulatory function of kisspeptin neurons on the secretion of GnRH. Furthermore, POMC neurons have direct synaptic connections with GnRH neurons (39), and *α*-MSH affects LH secretion depending on the ovulatory cycle in women (40). Kisspeptin, which is a regulator of GnRH neurons, has bidirectional communication with POMC neurons and is implicated in energy metabolism (41). This highly complex system involved in the regulation of GnRH secretion is required for evolutionary fitness. Some effects of POMC deficiency on the HPG axis should not be very surprising. The data on pubertal development in patients with POMC deficiency so far is scarce, particularly in males. Two girls lacked normal pubertal development with lower gonadotropins, which was not reversed by setmelanotide (9, 11). Another girl was reported to have spontaneous earlier normal puberty with late normal timing of menarche (17), indicating slower progressing puberty; however, one of the *POMC* gene variants in this girl targeted only ACTH and *α*-MSH and not the other POMC-derived peptides. Low gonadotropins during anticipated mini-puberty were observed in a 2.5-month-old girl, indicating congenital hypogonadotropic hypogonadism (23). The data suggest the follow-up for possible hypogonadism in girls with POMC deficiency as reasonable. The only male-related data derives from our patient who underwent delayed, spontaneous puberty and achieved low normal testicular volume and scarce androgen-dependent hairiness in adolescence. The patient had noticed significant body hair growth and began shaving at age of 22, which was a year after setmelanotide treatment had commenced. At the age of 25, on therapy, he had fully developed body hair with normal levels of gonadotropins and testosterone. The positive effect of weight loss on HPG and testosterone concentration is well established. Whether, in our patient, it was a direct effect of setmelanotide treatment and/or indirect effect of weight loss remains to be determined.

Another curious phenomenon that came to be noticed in our review was a surprisingly high incidence of T1D. Of all 22 cases, there were three patients with T1D (18, 21), including our male patient, which indicates a possible pathogenetic association between the two diseases. The research on rodents has proven an association between melanocortin signaling pathways and insulin action where central stimulation of MC4R leads to inhibition of insulin secretion *via* the peripheral neuronal pathway (42). In contrast, in POMC deficiency, there is a complete absence of MC4R stimuli. In the presence of autoantibodies in two of the reported cases (18, 21), the autoimmune etiology is the more probable cause. The anti-inflammatory effects of melanocortins have been well established *in vitro* and *in vivo* (43). In this regard, it would be reasonable to expect more autoimmune occurrences; yet, apart from T1D there were no such reports in POMC deficient patients. However, given the young age of most reported patients, it is too early to draw any meaningful conclusions. Considering the severe obesity and overt clinical signs of insulin resistance in some reported cases, including our 4-year-old girl, it would be reasonable to anticipate the eventual occurrence of type 2 diabetes, that according to the published data at young ages, seems to be rare (3).

In the 22 POMC deficient patients reviewed here, 15 different pathogenic variants in the *POMC* gene were identified (**Table 1** and **Figure 5**). Except for a single missense variant that affects *α*-MSH and ACTH peptides (17), all other variants are null-variants abolishing at least *α*-MSH, ACTH, and all other C-terminal peptides. The most common variant identified in six unrelated patients of Caucasian ancestry (7, 17, 18, 20) is c.-11C>A, which introduces a premature translation signal and subsequent false translation (4). The variant c.133-2A>C affects a splice site of the third exon, which encodes POMC from the 45th amino acid onwards (21). The current genetic data suggest that *α*-MSH and ACTH deficiencies were sufficient for the main phenotypic features of the disease. The data on minor or less frequently reported phenotypes were too scarce to provide further estimations on the potential roles of other POMC-derived peptides.

The main limitation of our review is the small number of reported cases and particularly the young age of most reported patients, which prevented us from any firm conclusions on disease complications that could become evident in adolescence or adulthood.

## Conclusions

As case reports continue to accumulate, the POMC deficiency reveals to be a more complex endocrine disorder than initially perceived and goes beyond its characteristic triad of adrenal insufficiency, early onset obesity, and red hair. The high hierarchal position of POMC and the multitude of melanocortin receptors and ligands explain its involvement in many neuroendocrinological functions, particularly in energy management, but also in skin and hair pigmentation, reproductive function, and growth. The absence of POMC may be associated with additional endocrine dysfunctions at least in a subset of patients. However, the exact role of POMC and the derived peptides in these endocrine functions remains to be determined. Ours and previously published experiences advocate for careful endocrine follow-up of POMC deficient patients through childhood and adolescence. Further collection of data for estimating the natural course of the disease and/or therapeutic effects of setmelanotide on hypothalamic-pituitary functions is needed.

## Ethics Statement

Written informed consents using local consent forms were obtained from the patient 1 and parents of the patient 2 for the publication of any potentially identifiable images or data included in this article.

## Author Contributions

UG and NG contributed to the study concept and design. MD and JK performed the molecular genetic analysis and data analysis with interpretation. NG, NB, MZ, JS, and MA collected the clinical data. NG, UG, and MA analyzed data obtained by the systematic review. NG and MA drafted the paper. PK and TB reviewed and edited the paper. MA is the guarantor of this work and, as such, had full access to all the data in the study and takes responsibility for the integrity of the data and the accuracy of the data analysis. All authors contributed to the article and approved the submitted version.

## Funding

This work received the financial support from the Slovenian Research Agency (research core funding No. P3-0343).

## Conflict of Interest

The authors declare that the research was conducted in the absence of any commercial or financial relationships that could be construed as a potential conflict of interest.
